# Integrating multi-omics and network toxicology to identify FIS1 as a key target of environmental pollutants in male infertility

**DOI:** 10.3389/fcell.2026.1788805

**Published:** 2026-03-19

**Authors:** Qi Yu, Xi Wei, Jiang Shi, Wei Li, Yili Zhao, Qingtao Yang, Jun Qiao, Fa Sun, Tao Li

**Affiliations:** 1 Department of Urology, the Affiliated Hospital of Guizhou Medical University, Guiyang, China; 2 Department of Reproductive Center, Affiliated Hospital of Guizhou Medical University, Guiyang, China

**Keywords:** FIS1, male infertility, mitochondrial dysfunction, reverse network toxicology, summary-data-based mendelian randomization analysis

## Abstract

**Background:**

Male infertility (MI) impacts about one in seven couples globally, involving complex gene-environment interactions. Environmental pollutants may disrupt spermatogenesis via mitochondrial dysfunction, but key targets and mechanisms are unclear. Integrating multi-omics with computational toxicology offers a novel strategy to decipher these interactions.

**Methods:**

We integrated blood transcriptomics from MI patients, GWAS summary statistics, and QTL data (methylation, expression, protein). MI-related mitochondrial genes were identified through differential expression analysis, followed by enrichment and PPI network analysis. SMR approach was employed to assess genetic causality between molecular levels of genes and MI risk, using cis-QTLs as instrumental variables and applying the HEIDI test (p < 0.05) to distinguish pleiotropy from linkage. The candidate gene FIS1 was functionally validated *in vitro* by siRNA knockdown in GC-1 spg cells. Reverse network toxicology was then used to screen environmental pollutants potentially targeting FIS1, with candidate compounds selected from the CTD based on unidirectional inhibitory effects on FIS1, high bioaccumulation potential (Log P > 5), and predicted toxicity (mutagenicity, cytotoxicity, or endocrine disruption). Binding affinity was evaluated via molecular docking.

**Results:**

We identified 232 dysregulated mitochondrial genes in MI. SMR analysis revealed that FIS1 showed a consistent, significant protective association with MI risk across three molecular levels: DNA methylation (e.g., site cg19802458), gene expression, and plasma protein abundance. Clinical samples confirmed downregulated FIS1 expression in MI patients. *In vitro*, FIS1 knockdown in spermatogonial cells reduced mitochondrial membrane potential, elevated reactive oxygen species, decreased antioxidant enzyme activity, and significantly inhibited proliferation. Reverse toxicology screened six environmental pollutants predicted to target FIS1, including di (2-ethylhexyl) phthalate, bisphenol S, aflatoxin B1, and benzo [a]pyrene. Molecular docking confirmed stable binding of all six compounds to the FIS1 protein (ΔG < −5.0 kcal/mol), suggesting a direct mechanism for disrupting mitochondrial function.

**Conclusion:**

By integrating multi-omics and computational toxicology, this study validates FIS1’s causal protective role in male infertility, reveals its multi-level regulation, and predicts six targeting pollutants with preliminary experimental evidence. This framework offers new insights into gene-environment interactions and establishes a foundation for biomarker development and targeted interventions.

## Background

Male infertility accounts for approximately half of all infertility cases, representing a major global health challenge. It affects about one in seven couples of reproductive age worldwide, is prevalent across diverse populations, and is associated with various long-term health risks. This condition imposes a heavy burden on patients, families, and healthcare systems. However, its pathogenesis is not fully understood, and effective prevention strategies are lacking (Minhas et al., 2025; Choy and Eisenberg, 2018; Rowaiee et al., 2025). Current research indicates that the disease arises from complex multifactorial interactions between genetic susceptibility and environmental exposures. Environmental factors, particularly during critical windows of reproductive development, may exacerbate inherent genetic vulnerabilities, thereby driving disease onset (He et al., 2024; Li et al., 2024). A significant challenge is that most existing studies focus on single substances, whereas real-world exposure involves complex mixtures of chemicals that may produce additive or synergistic toxic effects, posing considerable difficulties for traditional toxicological assessment (Li et al., 2024; Chai et al., 2021). Consequently, identifying key environmental exposure combinations and elucidating their molecular targets is of central importance for understanding the pathogenesis of male infertility and formulating evidence-based public health policies.

To address these gaps, integrative computational strategies are gaining increasing recognition (Qi et al., 2025; Wei et al., 2025; Xu et al., 2025). Summary-data-based Mendelian randomization (SMR) analysis can reveal how genetic variants influence disease initiation and progression by regulating molecular phenotypes such as DNA methylation, gene expression, and protein abundance, thereby systematically identifying potential disease-causing genes (Li et al., 2023). Furthermore, integrating this with reverse network toxicology methods allows for predicting environmental exposures that target these disease-related genes or pathways and for assessing the toxic potential of these environmental chemicals (Xu et al., 2025; Zhu et al., 2025). This integrated “gene-environment” analytical framework provides a powerful tool for in-depth analysis of the interplay between environmental exposure and genetic susceptibility in male infertility (Qi et al., 2025; Wei et al., 2025; Xu et al., 2025; Zhu et al., 2025).

Mitochondria, as the powerhouse of cellular energy metabolism, are integral to key biological processes including respiration, metabolism, signal transduction, and apoptosis (Wen et al., 2025). Their dysfunction can lead to insufficient cellular energy supply and elevated oxidative stress, thereby disrupting cellular homeostasis (Wen et al., 2025; Dominic et al., 2014). Recent studies highlight the crucial role of mitochondria in spermatogenesis; abnormalities in their structure and function can significantly disrupt sperm production, leading to male infertility (Boguenet et al., 2021; Wang et al., 2022; Nakada et al., 2006; Meng et al., 2025). Notably, mitochondria frequently serve as downstream targets for various environmental toxicants and are closely linked to pollution-related diseases, including male infertility (Lambertini and Byun, 2016; Caito and Aschner, 2015; Duarte-Hospital et al., 2021). For instance, studies have confirmed that exposure to environmental pollutants such as PM2.5, environmental cadmium, and low-dose bisphenol S can induce mitochondrial damage, leading to reproductive toxicity and spermatogenic dysfunction (Wang et al., 2024; Wang C. et al., 2025; Darghouthi et al., 2022), underscoring the pivotal role of mitochondria in environmentally associated male infertility.

To systematically elucidate these mechanisms, this study employs an integrated computational strategy. First, we identified differentially expressed mitochondrial-related genes in male infertility patients based on transcriptomic data. Subsequently, we utilized multi-omics data encompassing DNA methylation, gene expression, and protein abundance to construct instrumental variables for these genes. SMR analysis was then applied to identify mitochondrial regulatory genes genetically associated with male infertility, followed by experimental validation *in vitro*. Furthermore, by incorporating reverse network toxicology methods, we conducted molecular prediction and docking analyses on the identified genes. This approach aims to pinpoint environmental pollutants potentially causing infertility by interfering with mitochondrial function and to clarify their underlying mechanisms, thereby providing a novel theoretical basis for developing targeted, personalized therapeutic strategies.

## Methods and materials

### Data sources

The gene expression profiles related to MI were sourced from the Gene Expression Omnibus (GEO, http://www.ncbi.nlm.nih.gov/geo/) database, specifically datasets GSE145467 and GSE4797. In this study, GSE4797 was used for differential expression analysis to identify candidate genes associated with the disease state, while GSE145467 served as an independent validation set to confirm expression changes in the candidate genes. GWAS summary statistics for MI were obtained from the FinnGen Release 12, comprising 1,852 MI patients and 153,573 controls of European ancestry. MI cases were identified via diagnostic codes from ICD-8, -9, and -10 (including aspermia, oligospermia, testicular extirpation for infertility, and unspecified male infertility) (Kurki et al., 2023).

### Whole blood collection and processing

Peripheral venous blood samples were collected from NOA patients and healthy controls (5 NOA cases and five controls) using EDTA anticoagulant tubes. Whole blood was centrifuged at 3,000 × g for 10 min at 4 °C to separate plasma. According to the manufacturer’s instructions, the remaining blood cell pellet was immediately mixed with the Magen Blood RNA Kit (Guangzhou Magen Biotechnology Co., Ltd., Catalog No. R4161), vigorously vortexed, and stored at −80 °C until further processing.

### Acquisition of mitochondrial regulatory genes related to MI (MI-Mit)

We extracted a foundational set of 1,136 mitochondrial-associated genes from the comprehensive mitochondrial protein database MitoCarta3.0 (Rath et al., 2021). To identify genes related to MI, differential expression analysis was performed on the GSE145467 dataset using the ‘Limma’ package in R. The filtering thresholds were set at |log_2_FC| > 1 and adjusted p-value <0.05, yielding a list of genes significantly differentially expressed in MI patients. Finally, Venn analysis was used to identify the intersection between these differentially expressed genes and the mitochondrial gene set, resulting in a list of dysregulated mitochondrial regulatory genes in MI.

### Functional enrichment analysis

Gene Ontology (GO; http://geneontology.org) categorizes gene functions into Cellular Component (CC), Molecular Function (MF), and Biological Process (BP) domains. The Kyoto Encyclopedia of Genes and Genomes (KEGG; https://www.kegg.jp) systematically links genomic information to higher-level functional pathways. GO and KEGG enrichment analyses were performed using the R package clusterProfiler.

### Construction of protein-protein interaction (PPI) networks

The STRING database (confidence threshold ≥0.4) was used to construct PPI networks. After filtering out disconnected nodes, the data were imported into Cytoscape 3.9.0 for visualization. Topological parameters, including degree centrality, closeness centrality, and betweenness centrality, were calculated using the Centiscape 2.0 plugin. Nodes were ranked by importance based on closeness centrality, with higher values indicating a more central role in the network.

### Summary statistics for eQTL, mQTL, and pQTL data

The quantitative trait locus (QTL) data used in this study were sourced as follows: gene expression QTL (eQTL) data were from the eQTLGen Consortium, which integrated 37 studies with 31,684 samples, covering 10,317 trait-associated SNPs (Võsa et al., 2021); DNA methylation QTL (mQTL) data were cited from McRae et al.'s study based on two European cohorts (totaling 1,980 samples) (McRae et al., 2018); plasma protein QTL (pQTL) data were sourced from the deCODE team’s proteome-wide GWAS of 35,559 Icelandic individuals, which identified 18,084 variants associated with plasma protein levels, covering 257,490 associations (Ferkingstad et al., 2021). All QTL data underwent uniform preprocessing: first, genome-wide significant (p < 5 × 10^−8^) genetic variants were screened and annotated; they were then matched with the identified MI-Mit genes. Only MI-Mit targets that met the following criteria were retained for subsequent analysis: being located at the core of the MI-Mit interaction network and having valid signals in all three QTL datasets (mQTL, eQTL, and pQTL). This ensured the analysis was based on high-confidence genetic regulatory evidence.

### Summary-data-based mendelian randomization (SMR)

In this study, we employed the SMR method to assess the potential causal relationship between the DNA methylation, expression levels, and protein abundance of MI-Mit genes and MI. When summary data for both the exposure (e.g., QTL) and the outcome (MI) are derived from large-scale independent samples, SMR analysis based on significant cis-QTLs (top cis-QTL) generally offers greater statistical power than traditional Mendelian Randomization methods (Li et al., 2023). Concurrently, to distinguish pleiotropy from linkage effects, we performed the HEIDI test. A test p-value less than 0.05 suggests an association driven by linkage disequilibrium; such results were considered not to meet the assumptions for causal inference and were excluded from subsequent analysis (Zhu et al., 2016). The linkage disequilibrium reference data used in the analysis were obtained from the 1000 Genomes Project. The SMR analysis and HEIDI test described above were conducted using the SMR software (version 1.3.1, available at: https://yanglab.westlake.edu.cn) developed and maintained by the Yang lab.

### Cell culture and treatment

The GC-1 spg (ts) mouse spermatogonial cell line was purchased from the Cell Bank of the Chinese Academy of Sciences. Cells were cultured at 37 °C in a humidified incubator with 5% CO_2_, following the supplier’s protocol. To investigate the role of the FIS1 gene in these cells, we downregulated its expression using RNA interference. Targeted small interfering RNA (siRNA) against FIS1 was transfected into GC-1 spg (ts) cells using Lipofectamine 8000 (Beyotime Biotechnology, China) and Opti-MEM medium (Gibco, USA). The siRNA sequences used in the experiments were synthesized by Sangon Biotech Co., Ltd. The details of the targeting sequence (si-FIS1) are as follows: Forward: 5′-CACCG GTT​GCC​CAA​AGG​GAG​CAA​AG-3′, Reverse: 5′-AAACC CTT​TGC​TCC​CTT​TGG​GCA​ACC-3’. To evaluate the effect of benzo(a)pyrene on GC-1 spg (ts) cells, based on previous literature reports, we applied the compound at a concentration of 30 μM for treatment.

### Western blotting

Total cellular protein was extracted using RIPA lysis buffer (Solarbio, China) supplemented with protease inhibitors (Yeasen, China). Protein concentration was determined using the Pierce™ BCA Protein Assay Kit (Thermo Fisher Scientific, USA). Equal amounts of protein samples were separated by electrophoresis on 8%–12% gradient SDS-PAGE gels. Proteins were then transferred onto PVDF membranes using a semi-dry transfer system (Bio-Rad, USA). Membranes were blocked at room temperature for 1 h with TBST containing 5% skim milk, followed by overnight incubation at 4 °C with the primary antibody (anti-FIS1, 1:7000, #10956-1-AP, Proteintech). After washing with TBST, membranes were incubated with HRP-conjugated secondary antibodies at room temperature for 2 h. Finally, protein bands were quantified using Image Lab™ software.

### RNA isolation and quantitative real-time PCR (qPCR)

Total RNA was extracted from whole blood samples using the Blood RNA Kit according to the manufacturer’s protocol, and RNA purity was verified on a NanoDrop 2000 spectrophotometer (acceptable A260/A280 ratio >1.8). High-quality RNA was reverse-transcribed into cDNA with the PrimeScript™ RT Reagent Kit. Quantitative PCR was then conducted on a QuantStudio five system using the Premix Ex Taq™ Kit under the following cycling conditions: initial denaturation at 95 °C for 30 s, followed by 40 cycles of 95 °C for 5 s and 60 °C for 34 s. All reactions were performed in triplicate. The primer sequences used were: Forward: 5′-GTA​GGG​TTA​CAT​GGA​TGC​CCA​GAG​A-3′, Reverse: 5′-GGC​AAA​AGC​TCC​TCC​AGC​AG-3′.

### Oxidative stress detection

Similarly, penile corpus cavernosum tissue extracts were used to measure reactive oxygen species (ROS), concentrations of malondialdehyde (MDA) (BC0025, Solarbio), and superoxide dismutase (SOD) (BC5165, Solarbio) to assess oxidative activity. The concentrations of the above indicators were normalized to their corresponding protein concentrations using the appropriate assay kits according to the manufacturer’s instructions.

### EdU proliferation assay

Cell proliferation of GC-1 spg (ts) cells was analyzed using the Cell-Light™ EdU Apollo® 488 *In Vitro* Imaging Kit (Beyotime, China). Cells were seeded in 24-well plates at a density of 3 × 10^4^ cells per well and cultured until 70%–80% confluent. Cells were treated with 20 μM EdU working solution (Servicebio, China) for 2 h at 37 °C with 5% CO_2_. Subsequently, cells were fixed with 4% paraformaldehyde for 15 min and permeabilized with 0.5% Triton X-100 for 20 min. Following the kit instructions, proliferating cells that had incorporated EdU were fluorescently labeled using the Click-iT™ EdU Alexa Fluor™ 594 Imaging Kit (Thermo Fisher Scientific, USA). Finally, fluorescent images were captured using a Nikon Eclipse Ts2R FL inverted fluorescence microscope at 20× magnification.

### Environmental pollutant prediction

To identify environmental pollutants that could potentially regulate the core causal gene FIS1, this study queried the Comparative Toxicogenomics Database (CTD) (Davis et al., 2023). When screening candidate pollutants, the following criteria were applied to ensure biological relevance and specificity: Only compounds documented in the CTD with clear, unidirectional regulatory effects (inhibition) were retained for analysis. Conversely, compounds reported to have conflicting or bidirectional (activating and inhibitory) effects under different experimental conditions, or those influencing FIS1 expression only through indirect pathways, were excluded from further analysis.

### Toxicological property prediction

To systematically assess the toxicological characteristics of candidate compounds, this study utilized two specialized computational platforms: the ADMETlab 3.0 platform (Fu et al., 2024) was used to predict absorption, distribution, metabolism, and excretion properties, and the ProTox-III platform (Banerjee et al., 2024) was used for multi-endpoint toxicity assessment. The screening process focused on the compounds' bioaccumulation potential, mutagenicity, cytotoxicity, and endocrine-disrupting activity. Ultimately, compounds exhibiting clear bioaccumulation potential and a positive prediction for at least one of the other three toxicity endpoints were selected for in-depth analysis.

### Molecular docking

After screening for environmental pollutants with a clear regulatory relationship, molecular docking simulations were performed to further validate their interaction with the target protein. Two-dimensional molecular structures of the environmental pollutants and natural products were retrieved from the PubChem database, while the three-dimensional structures of key target proteins were sourced from the AlphaFold Protein Structure Database. AutoDockTools 1.5.7 software was used to prepare the ligand and receptor structures and to perform molecular docking simulations, predicting binding modes, calculating binding free energy (ΔG), and assessing potential functional impacts. A binding free energy below 0 kcal/mol indicates a spontaneous binding process, while a value below −5.0 kcal/mol suggests a stable interaction.

### Statistical analysis

All statistical analyses were performed using R software (version 4.2.2). Differences in gene expression were assessed using the non-parametric Wilcoxon signed-rank test and the parametric paired Student’s t-test, with statistical significance set at a two-sided p-value <0.05. All *in vitro* experiments were performed at least three independent times.

## Results

### Identification and enrichment analysis of MI-related mitochondrial genes

**FIGURE 1:**
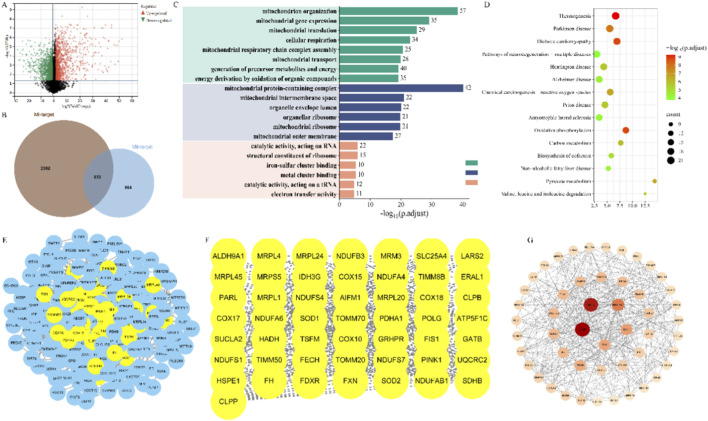
Acquisition and enrichment analysis of dysregulated mitochondrial regulatory genes and construction of PPI network in NOA patients. **(A)** Volcanic map of differentially expressed genes in NOA patients; **(B)** Acquisition of mitochondrial related genes dysregulated in NOA patients; **(C)** GO enrichment analysis; **(D)** KEGG enrichment analysis; **(E–G)** Construction of PPI network and screening process of core genes.

Differential expression analysis of blood samples from MI patients identified a total of 2,594 differentially expressed genes, comprising 1,191 upregulated and 1,403 downregulated genes (Figure 1A). Subsequent Venn analysis identified 232 overlapping genes common to both the differentially expressed gene set and the mitochondrial gene set (Figure 1B). These genes are considered potential mitochondrial regulators related to MI pathogenesis. GO enrichment analysis indicated that these genes are primarily involved in mitochondrial regulation, oxidative phosphorylation, and various metabolic processes (Figure 1C). Further KEGG pathway analysis revealed that they are also closely associated with the development of multiple chronic diseases and related metabolic pathways (Figure 1D).

### Protein-protein interaction network construction

The 232 overlapping MI-Mit genes were imported into the STRING database for PPI analysis with a confidence threshold set to ≥0.4. After removing isolated nodes, a final interaction network comprising 217 target proteins was obtained. Cytoscape 3.10.3 was used for network visualization, with node size and color intensity mapped based on their degree of connectivity (higher connectivity corresponds to larger diameter and darker color). Topological analysis further identified the top 50 core targets with the highest connectivity within the network, with CLPP, SDHB, NDUFAB1, SOD2, and FXN ranking in the top five (Figure 1E/F/G). This network visualization not only clearly illustrates the interactions among key targets but also provides important clues for further exploring the potential molecular mechanisms of mitochondrial involvement in male infertility.

### Genetic susceptibility to male infertility via DNA methylation of mitochondrial genes

**FIGURE 2:**
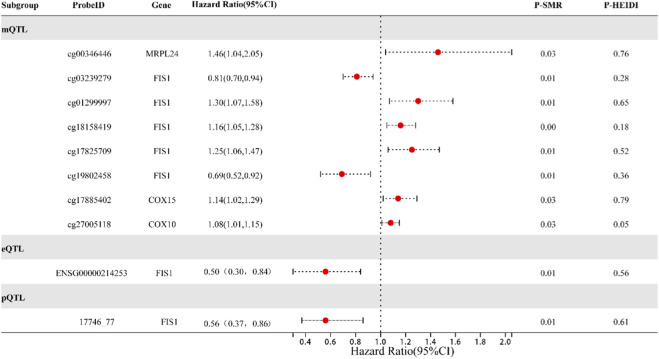
mQTL, eQTL, and pQTL of Mitochondrial-Related Genes with Causal Relationships to MI Risk. Note: This figure summarizes the results of summary-data Mendelian randomization (SMR) analysis investigating the causal associations between molecular levels of mitochondrial-related genes and male infertility (MI) risk. Each point represents a specific CpG site (mQTL), gene expression level (eQTL), or protein abundance (pQTL) instrumented by cis-QTLs. The x-axis indicates the odds ratio (OR) for MI risk, with error bars representing 95% confidence intervals. All displayed loci passed the HEIDI test (p > 0.05), indicating that associations are not driven by linkage disequilibrium.

Following screening, 29 genes located at the core of the MI-Mit interaction network were found to have valid signals in the mQTL data. These genes corresponded to 66 relevant CpG sites. After SMR analysis and the HEIDI test, four sites were excluded due to a HEIDI test p-value <0.05 or invalid signals. Among the remaining 62 CpG sites, eight were significantly associated with MI risk (Figure 2). Specifically, cg19802458 (FIS1) and cg03239279 (FIS1) were negatively correlated with MI risk, whereas cg27005118 (COX10), cg17885402 (COX15), cg18158419 (FIS1), cg17825709 (FIS1), cg01299997 (FIS1), and cg00346446 (MRPL24) showed a positive correlation.

### Association of FIS1 expression and protein levels with male infertility risk

After screening, 11 genes located at the core of the MI-Mit interaction network were found to have valid genetic signals in the eQTL data. SMR and HEIDI test analysis excluded two genes (MRPL1 and FECH) that failed the HEIDI test. Among the remaining nine genes, only FIS1 demonstrated a potential causal association with MI risk (OR = 0.50; 95% CI: 0.30–0.84, p-value = 0.009) (Figure 2).

Through screening, four genes located at the core of the MI-mitochondrial interaction network showed significant signals in protein quantitative trait locus (pQTL) data. SMR and HEDI analyses revealed that only FIS1 demonstrated a potential causal relationship with myocardial infarction risk (OR = 0.56; 95% CI: 0.37–0.86, p = 0.008) (Figure 2). In summary, FIS1 was significantly associated with MI risk at three molecular phenotypic levels, DNA methylation, gene expression, and protein abundance, and occupied a central position in the MI-mitochondrial interaction network. Therefore, this study identified FIS1 as a key disease-associated gene related to mitochondrial function in MI and designated it as the core target for subsequent in-depth investigation.

### Multi-omics regulatory landscape of FIS1

**FIGURE 3:**
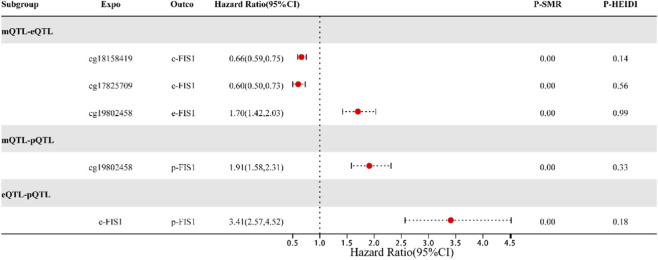
Regulatory Relationships among mQTL, eQTL, and pQTL of the FIS1 Gene. Note:This figure illustrates the causal relationships between DNA methylation, gene expression, and protein abundance of FIS1, as revealed by SMR analysis based on shared genetic variants. Each arrow represents a significant association (p < 0.05) passing the HEIDI test, indicating that the relationship is not driven by linkage disequilibrium.

Based on shared genetic variants, this study employed SMR analysis to systematically explore the causal relationships among methylation, gene expression, and protein levels of mitochondrial-related genes. The results (Figure 3) showed that for the FIS1 gene, increased methylation at CpG sites cg18158419 and cg17825709 was significantly associated with decreased blood expression, whereas methylation at cg19802458 was associated with increased FIS1 expression and protein levels. Concurrently, FIS1 blood expression levels were positively correlated with its protein abundance. Integrating these findings with the earlier independent conclusion that “higher FIS1 levels are associated with lower MI risk,” this multi-omics evidence collectively suggests that FIS1 may play a protective role in MI. Different methylation sites within FIS1 may differentially regulate its gene expression and protein abundance, thereby exerting varying directional effects on MI risk.

### Validation of FIS1 expression in clinical samples and public datasets

**FIGURE 4:**
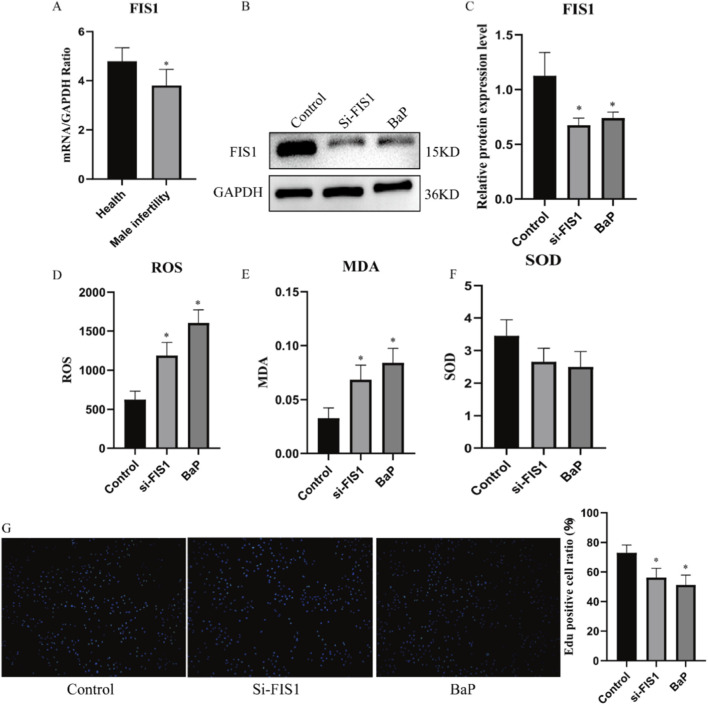
The Protective Effect of the FIS1 Gene on GC-1-spg (ts) Cells and the Inhibitory Effect of BaP on GC-1-spg (ts) Cells; **(A)** mRNA levels of FIS1 in the blood of MI patients; **(B)** Changes in FIS1 protein levels in GC1 spg (ts) cells after FIS1 knockdown and BaP intervention; **(D-F)**: The effect of FIS1 knockdown and BaP intervention on the oxidative balance of GC one spg (ts) cells, **(D)** ROS, **(E)** MDA, **(C)** SOD; **(G)** The effect of FIS1 knockdown and BaP intervention on the proliferation of GC1 spg (ts) cells.

First, based on two independent transcriptomic cohorts (GSE145467 and GSE4797), FIS1 expression was found to be downregulated in the blood of MI patients (Supplementary Figures S1A,B). Subsequently, immunohistochemical results from testicular tissue in the Human Protein Atlas (HPA) database showed moderate FIS1 protein expression throughout the testis, including in spermatogenic cells and Leydig cells (Supplementary Figure S1C). Similarly, single-cell data documented in the HPA database indicated that FIS1 is expressed across all cell populations, with the highest expression levels observed in late spermatids (Supplementary Figure S1D). Furthermore, qPCR validation using clinical blood samples confirmed a significant decrease in FIS1 mRNA levels in MI patients (Figure 4A). In summary, these expression trends, consistent with the direction of the SMR analysis, reinforce that downregulation of FIS1 is closely associated with the occurrence of MI.

### Protective role of FIS1 in GC-1 spg cells: *In Vitro* functional validation

First. In the GC-1 spg (ts) cell, Western blot results further confirmed a reduction in FIS1 protein levels (Figure 4B), indicating successful genetic interference. ELISA assays showed that decreased FIS1 expression led to elevated levels of pro-oxidant factors ROS and MDA, alongside reduced activity of the antioxidant enzyme SOD (Figures 4C-E), suggesting a state of oxidative stress, often associated with mitochondrial dysfunction. Additionally, EdU assays demonstrated that FIS1 downregulation significantly inhibited the proliferation of GC-1 spg (ts) cells (Figure 4F). Collectively, these findings indicate that FIS1 exerts a protective role in GC-1 spg (ts) cells, further supporting its function as a protective factor in the pathogenesis of MI.

### Screening and molecular docking of environmental pollutants targeting FIS1

**FIGURE 5:**
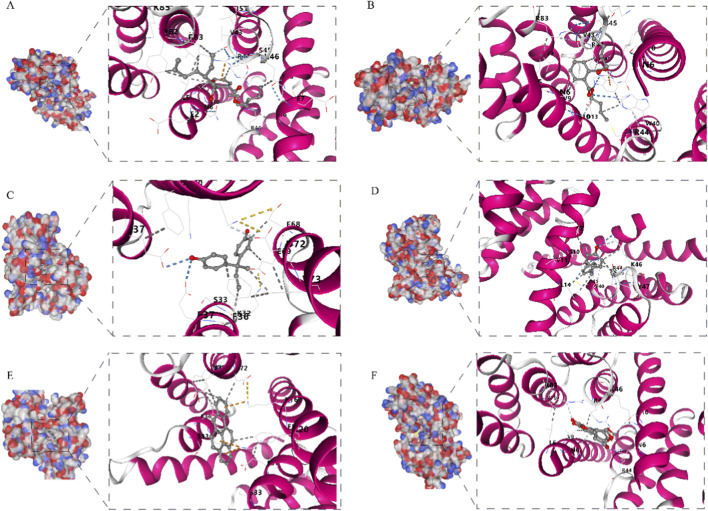
Molecular Docking Results of Six Environmental Pollutants Capable of Stably Binding to FIS1; **(A)** Di (2-ethylhexyl) phthalate, **(B)** dibutyl phthalate, **(C)** bisphenol B, **(D)** bisphenol S, **(E)** aflatoxin B1, and **(F)** benzo (a) pyrene.

Initial screening identified nine common environmental pollutants: dimethyl phthalate, di (2-ethylhexyl) phthalate (DEHP), dibutyl phthalate (DBP), bisphenol A (BPA), bisphenol B (BPB), bisphenol S (BPF), aflatoxin B1, benzo [a]pyrene (BaP), and air pollutants (specifically benzene, sulfur dioxide, carbon monoxide, toluene, and ozone). These were subsequently filtered based on pre-defined criteria: dimethyl phthalate and the air pollutant mixture were excluded due to low lipophilicity (Log P < 5, suggesting weak bioaccumulation potential); BPA was excluded because it exhibited bidirectional or inconsistent effects on FIS1 expression across different studies. The remaining six pollutants met the following criteria: the ability to regulate FIS1 expression, high bioaccumulation potential (Log P > 5), and positive predictions for at least one toxicity endpoint (e.g., mutagenicity, cytotoxicity, or endocrine disruption) in toxicological assessments. Further molecular docking analysis revealed that all six compounds could bind stably to the FIS1 protein. Their binding free energies (ΔG) were as follows: DEHP (−5.9 kcal/mol; Figure 5A), DBP (−6.4 kcal/mol; Figure 5B), BPB (−6.3 kcal/mol; Figure 5C), BPF (−6.3 kcal/mol; Figure 5D), aflatoxin B1 (−7.4 kcal/mol; Figure 5E), and BaP (−7.1 kcal/mol; Figure 5F).

### *In Vitro* validation of Benzo [a]pyrene-Induced FIS1 downregulation and cellular damage

Western blot analysis confirmed that BaP intervention reduced FIS1 protein levels in GC-1 spg (ts) cells (Figure 4B). ELISA assays showed that BaP exposure increased intracellular levels of pro-oxidant factors (ROS and MDA) and decreased antioxidant enzyme (SOD) activity (Figures 4C-E), indicating a state of oxidative stress. In addition, EdU assays further revealed that BaP significantly suppressed the proliferation of GC-1 spg (ts) cells. Together, these results demonstrate that BaP exerts damaging effects on GC-1 spg (ts) cells and highlight the key role of reduced FIS1 expression in mediating these effects.

## Discussion

Male infertility is a significant health issue affecting approximately one in seven couples of reproductive age worldwide. Its pathogenesis is complex, involving interactions between genetic susceptibility and environmental exposures (Minhas et al., 2025; Choy and Eisenberg, 2018; Rowaiee et al., 2025; He et al., 2024; Li et al., 2024). Environmental factors often exist as chemical mixtures in real life, posing challenges for elucidating their pathogenic mechanisms (Li et al., 2024; Chai et al., 2021). Mitochondria, serving as the energy and metabolic hubs during spermatogenesis, are often key targets of environmental toxicants. Their dysfunction is closely linked to male infertility (Wen et al., 2025; Dominic et al., 2014; Boguenet et al., 2021; Wang et al., 2022; Nakada et al., 2006; Meng et al., 2025; Lambertini and Byun, 2016; Caito and Aschner, 2015; Duarte-Hospital et al., 2021). Although it is known that genetic and environmental factors collectively drive disease onset, the specific interactive mechanisms on particular biological pathways, such as mitochondrial function, remain incompletely understood. This is especially true regarding how they “program” long-term health outcomes during critical windows of reproductive development. Similarly, while mitochondria are recognized as downstream targets of environmental toxicants, the key molecular events and regulatory nodes within the causal chain from specific environmental exposure to mitochondrial dysfunction and subsequently to impaired spermatogenesis await systematic identification and validation.

To address this, based on existing literature and transcriptomic data from MI patients, we identified 232 differentially expressed mitochondrial-related genes in MI. By further integrating protein-protein interaction networks with multi-omics data encompassing methylation, transcriptomics, and proteomics, we systematically identified mitochondrial genes associated with genetic susceptibility to MI. The results indicated that the methylation level, gene expression, and serum protein abundance of FIS1 were all associated with MI risk. Validation using clinical samples showed a significant reduction in FIS1 mRNA levels in the blood of MI patients. Furthermore, *in vitro* experiments based on gene interference confirmed the protective role of FIS1 in GC-1 spg (ts) mouse spermatogonial cells, further supporting its key role in spermatogenesis. Mechanistically, we found that increased methylation at the cg19802458 site promoted higher FIS1 expression and protein abundance, thereby reducing MI risk, whereas increased methylation at the cg18158419 and cg17825709 sites might increase MI risk by suppressing FIS1 expression. This finding is consistent with Chen et al.'s conclusion that cg19802458 positively regulates FIS1 expression (Chen et al., 2024). In summary, this study, for the first time based on population multi-omics data, reveals a causal association between FIS1 and reduced MI risk and preliminarily clarifies the related epigenetic regulatory pathways, providing new clues for understanding the molecular mechanisms of MI.

The FIS1 gene encodes the Mitochondrial Fission 1 Protein, a key regulator of mitochondrial fission (Cerveny et al., 2007). This process is crucial for maintaining mitochondrial quantity, dynamic morphology, cellular function, and survival (Cerveny et al., 2007; Losón et al., 2013; Zhao et al., 2024) and plays an important role in maintaining cellular homeostasis and resisting oxidative stress (Pokhrel et al., 2025; Rovira-Llopis et al., 2017; Dykstra et al., 2021). Mitochondrial dysfunction and oxidative stress are both closely associated with the occurrence of MI (Boguenet et al., 2021; Wang et al., 2022; Nakada et al., 2006; Meng et al., 2025). Although current population epidemiological and mechanistic evidence directly linking FIS1 to MI remains limited, animal model studies suggest an important role for FIS1 in testicular functional homeostasis. For example, Liu et al. (Liu et al., 2024) found decreased FIS1 expression in the testes of aging male mice; Varuzhanyan et al. (Varuzhanyan et al., 2021) confirmed through gene knockout that FIS1 deficiency in the male germline of mice leads to early spermatogenic arrest accompanied by increased mitochondrial content. Some studies have also noted decreased FIS1 expression in models of testicular dysfunction induced by gut metabolites and environmental toxicants (Wang S. et al., 2025; Jin et al., 2025). These findings align with the direction of our results, supporting the critical role of FIS1 in reproductive health.

Furthermore, based on CTD database mining and reverse network toxicology analysis, this study predicted six environmental pollutants potentially targeting FIS1. Molecular docking simulations revealed that these compounds can stably bind to the FIS1 protein, providing preliminary clues from computational toxicology that they may affect mitochondrial function through direct action on FIS1. Notably, although these pollutants have been reported in animal studies to be associated with reduced male fertility (He et al., 2024; Li et al., 2024), direct evidence linking them to FIS1 and extrapolating to human male infertility is still lacking, with critical population-based exposure-disease association evidence absent. This study preliminarily validated the downregulatory effect of BaP on FIS1 and its damaging effects on spermatogonial cells through *in vitro* experiments; however, these findings require further confirmation in broader experimental systems (such as dose-response relationships for multiple compounds, primary cells, or animal models).

It is worth discussing that the association between peripheral blood FIS1 expression levels (systemic level) and testicular spermatogenic dysfunction (local alteration) can be reasonably explained from the following perspectives. First, due to the difficulty in obtaining testicular tissue samples, large-scale multi-omics GWAS data from testicular tissue are currently lacking. Therefore, this study employed large-sample, easily accessible peripheral blood multi-omics data as an alternative strategy to investigate the association between FIS1 and male infertility. Despite tissue specificity, FIS1, as a key protein involved in mitochondrial fission, exhibits expression changes that reflect systemic mitochondrial functional status. Given that spermatogenesis is highly dependent on mitochondrial energy metabolism, downregulation of FIS1 in peripheral blood may indirectly indicate impaired overall mitochondrial health, thereby affecting testicular spermatogenesis.

Second, environmental pollutants (such as benzo [a]pyrene and phthalates) absorbed into the systemic circulation can simultaneously act on blood cells and germ cells. Therefore, peripheral blood FIS1 may serve as a “sentinel” biomarker capturing the cumulative effects of environmental exposure, reflecting exposure-induced mitochondrial damage burden. This perspective provides new insights into understanding how environmental factors influence reproductive health through systemic pathways. Furthermore, substantial evidence supports the use of peripheral blood transcriptomes as proxy indicators for inaccessible tissues. In this study, the consistency of the protective trend of FIS1 between peripheral blood and testicular tissue further strengthens the biological plausibility of this association. In conclusion, despite limitations related to tissue specificity and sample source, peripheral blood FIS1, as a comprehensive reflection of systemic mitochondrial health, environmental exposure burden, and potential inter-tissue communication, holds significant biomarker value in male infertility risk assessment.

Compared with previous studies (Varuzhanyan et al., 2021), our work provides several unique added values. First, through integrated multi-omics SMR analysis, we establish for the first time at the population genetic level the causal associations between FIS1 DNA methylation (e.g., cg19802458), gene expression, and protein abundance with MI risk, thereby extending animal model findings to humans. The SMR method itself reduces confounding and excludes reverse causality, strengthening causal inference. Second, we systematically reveal the multi-directional regulatory relationships among different CpG sites of FIS1 on its expression and protein levels, offering new insights into its epigenetic regulatory mechanisms. Third, using reverse network toxicology, we predicted six environmental pollutants (e.g., phthalates, bisphenols, benzo [a]pyrene) that may target FIS1, and via molecular docking and *in vitro* experiments we validated that benzo [a]pyrene downregulates FIS1 and induces spermatogonial cell damage. This integration of computational predictions with experimental validation not only provides a novel mechanistic pathway linking environmental exposures to mitochondrial gene disruption but also opens avenues for discovering early biomarkers and designing targeted interventions. Thus, rather than merely replicating animal findings, our study systematically expands and deepens the understanding of FIS1 in male infertility from three dimensions: population genetics, epigenetic regulation, and environmental interactions.

While this study provides valuable insights into the role of FIS1 in male infertility and its potential as a target of environmental pollutants, several limitations should be considered when interpreting the generalizability of our findings. First, the genetic and multi-omics data used in this study—including GWAS summary statistics from FinnGen and QTL data from European cohorts—are primarily derived from populations of European ancestry. This may limit the applicability of our conclusions to other ethnic groups, as genetic architecture, allele frequencies, and epigenetic regulation can vary significantly across populations. Future studies should aim to validate the causal relationship between FIS1 and male infertility in diverse ethnic cohorts to confirm the universal relevance of our findings.

Second, the assessment of environmental pollutants potentially targeting FIS1 was based on the CTD and computational predictions (e.g., molecular docking), rather than on individualized exposure measurements. While this approach provides a valuable framework for hypothesis generation, it cannot fully capture the complexity of real-world mixed exposures, including potential synergistic or antagonistic effects among chemicals. Moreover, the lack of individual-level exposure data limits our ability to draw definitive conclusions about the specific pollutants that may contribute to male infertility in different environmental contexts. Future research should incorporate prospective cohort studies with detailed exposure assessments and biomonitoring to validate the predicted pollutant–FIS1 interactions.

Finally, this study has a tissue specificity mismatch between the mQTL data (derived from peripheral blood) and the functional validation model (GC-1 spg cells). DNA methylation patterns are highly tissue-specific, and the regulatory relationships observed in blood cannot be directly extrapolated to testicular germ cells. Future studies should aim to validate the methylation-regulatory relationships of FIS1 directly in human testicular tissue or isolated germ cells using targeted bisulfite sequencing or single-cell methylation profiling. Additionally, the development of testis-specific mQTL datasets from large cohorts would enable more accurate tissue-matched SMR analysis. Advanced models such as human induced pluripotent stem cell (iPSC)-derived testicular organoids could also be employed to study the functional consequences of specific methylation changes in a human-relevant context.

Despite these limitations, this study establishes an innovative gene-environment interaction research framework by integrating multi-omics analysis, SMR, and reverse network toxicology, laying a theoretical foundation and identifying key directions for future research on male infertility. First, the consistent association of FIS1 methylation, expression, and protein levels with male infertility risk suggests that its related molecular signatures hold promise as biomarkers for early diagnosis or risk stratification, warranting further validation in large-scale prospective cohorts. Second, the environmental pollutants identified in this study (e.g., DEHP, bisphenols, and BaP) provide a basis for developing targeted public health intervention strategies, particularly in reducing exposure to these chemicals in susceptible populations. Furthermore, future research should employ advanced models such as CRISPR-based gene editing and human induced pluripotent stem cell (iPSC)-derived testicular organoids to elucidate the precise molecular mechanisms underlying the interactions between environmental pollutants and FIS1. Finally, subsequent studies should expand the analytical scope from mitochondrial genes to other key pathways, including autophagy, DNA repair, and endocrine signaling, to construct a more comprehensive gene-environment interaction network in male infertility. Advancing these directions will facilitate the translation of current findings into clinical applications and evidence-based public health policies.

## Conclusion

By integrating multi-omics and computational toxicology approaches, this study validated the causal protective role of FIS1 against male infertility at the population genetic level and revealed its multi-layered regulatory characteristics involving methylation, expression, and protein levels. Despite the tissue specificity differences in methylation regulation between blood and testicular tissue, the functional validation results of FIS1, as a key mitochondrial fission protein, in spermatogonial cells were highly consistent with the population genetic findings, collectively supporting its importance in male infertility. The integrated research framework constructed in this study provides a new perspective for understanding gene-environment interactions in male infertility and establishes a theoretical foundation for subsequent biomarker development and targeted intervention strategies.

## Data Availability

The original contributions presented in the study are included in the article/Supplementary Material, further inquiries can be directed to the corresponding authors.
