# Supplementary material for: Integrating multi-omics and network toxicology to identify FIS1 as a key target of environmental pollutants in male infertility
Source: Front Cell Dev Biol. 2026 Mar 19;14:1788805. doi: 10.3389/fcell.2026.1788805 (PMC13044088; doi:10.3389/fcell.2026.1788805)
Supplement: Supplementary file 1 [file DataSheet1.docx]

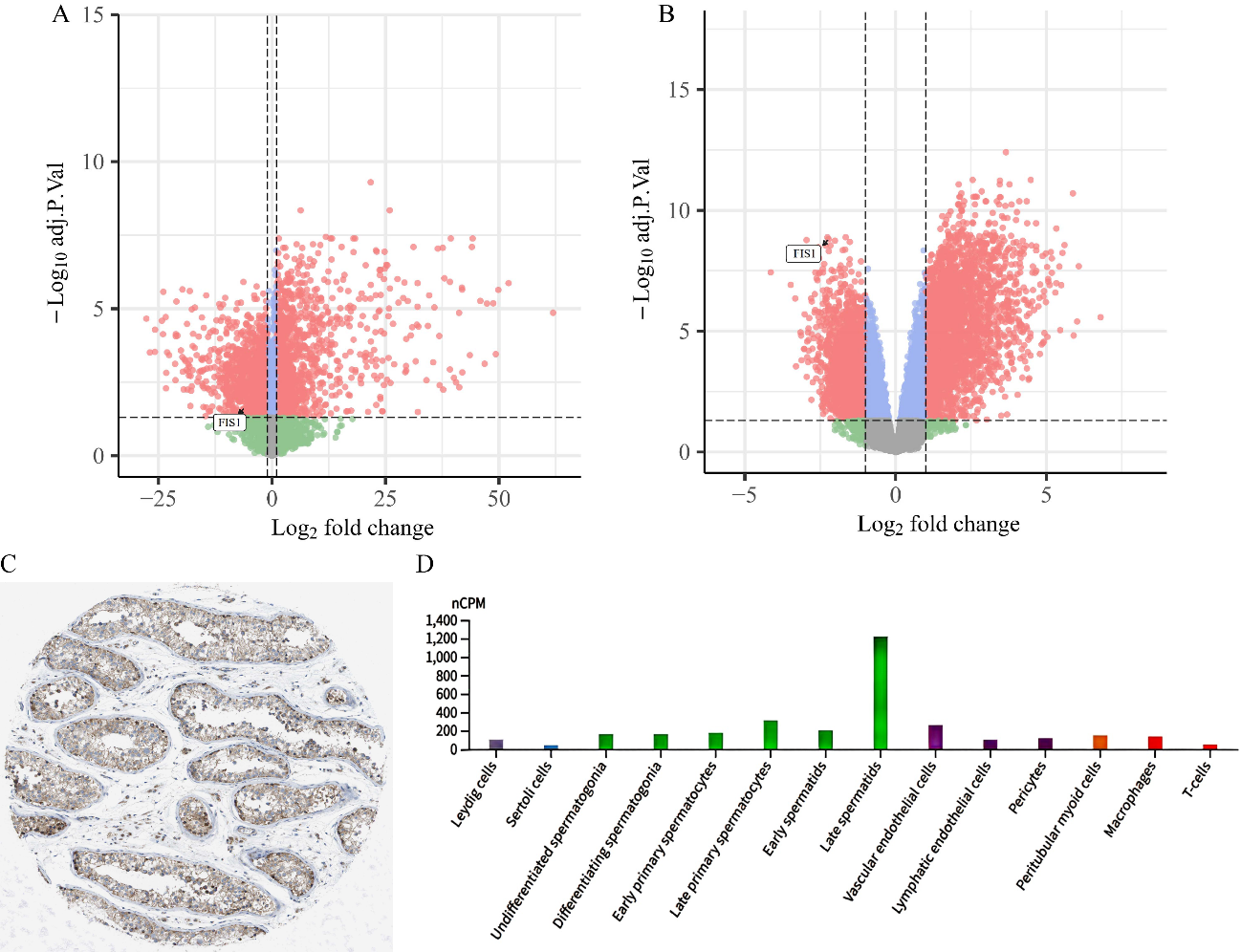


Figure S1: Expression validation and single-cell localization of FIS1

A-B: Transcriptome levels of FIS1 in two independent cohorts, A: GSE4797 queue, B: GSE145467 queue; C: Immunohistochemistry of FIS1 in testicular tissue recorded in HPA database; D: The expression of FIS1 recorded in the HPA database in single-cell data clustering of testicular tissue
